# A comprehensive evaluation of regional carbon emission based on the composite model in China: a case study of Huaibei city (China)

**DOI:** 10.1038/s41598-023-42667-0

**Published:** 2023-09-16

**Authors:** Zheng Wen, Ziwei Yang, Qinfeng Xing

**Affiliations:** State Key Laboratory of Mining Response and Disaster Prevention and Control in Deep Coal Mines, Anhui University of Science and Technology, Huainan, 232001 Anhui China

**Keywords:** Environmental sciences, Environmental social sciences

## Abstract

Formulating carbon emission reduction at the regional level is key to achieving the “dual carbon” strategy. A composite model is used to analyze the carbon emission reduction in Huaibei City based on the data from 2012 to 2021 and predict its change trend of carbon emission from 2022 to 2030. The study finds that: (1) the effects of observed factors on carbon emission in Huaibei City are complicated. Among them, the secondary industry has the greatest impact on carbon emission (weight is 0.32), and it is the key constraint factor of carbon emission. Population has the smallest impact on carbon emission (weight is 0.13), but its obstacle effect is significant. (2) The observed factors have a significant positive effect on the “dual carbon” strategy, but the improvement pressure is high. Among them, the conditions of all indicators have been improved except population, and it is found that each indicator can reach the level I before 2030, the “dual carbon” strategy can be achieved. This study deepens the understanding of regional carbon emission in China and the following conclusions are formed: (1) Grasping the carbon reduction effect of urbanization on the population to consolidate the new model of low-carbon development. (2) Identifying the key areas of carbon emission reduction to build a low-carbon emission oriented industrial system. (3) Strengthening the technological innovation of carbon emission reduction to achieve the strategic goal of “dual-carbon”.

## Introduction

Achieving the strategic goal of “dual-carbon” is not only a systematic economic and social reform but also a new revolution of low-carbon development^1–3^. According to the actual situation, the realization of “dual carbon” strategy should be decomposed by the principle of “common but differentiated responsibilities” at the regional level^4^. As to this question, how to identify the deduction processes of carbon emission and solve the problem of “common but differentiated responsibilities” is not only an important practical problem, but also an academic problem.

In the academic field, scholars have conducted corresponding research on the topic of “carbon emission”, mainly in the selection and system construction of carbon emission influencing factors, the methods of evaluation and prediction on carbon emission, and the implementation path of carbon emission reduction. Furthermore, for the selection of influencing factors, mainly evaluation indicators are selected from economic, energy, and industrial^5–7^. Specifically, it was found that population and economic growth are the main driving factors of carbon emission, and industrial structure rationalization and technological innovation are positive factors in the process of carbon emission reduction^8,9^. Moreover, for the methods of evaluation and prediction on carbon emission. STIRPAT topology model, OLS ridge regression model and LEAP model were often used to assess the development of carbon emission in the past^10–13^, and BP neural network model and gray prediction model were developed to predict the change trend of carbon emission in the future^14,15^. Combined with the results of carbon emission level assessment and identification the development trend of carbon emission in the future, it can provide effective guidance for the implementation path of carbon emission reduction. Additionally, for the regulation mechanism of carbon emission reduction, some scholars suggested that low-carbon system development and technological innovation can have a fluctuating impact on green economic growth, which in turn reduces carbon emission^16–19^. Meanwhile, a hybrid governance system integrating multiple carbon emission regulatory tools should be designed as a supplement to provide institutional mechanisms to regulate the trading market of carbon emission through the guarantee of law-moral governance^20–23^. Existing studies have made great contributions to the field of carbon emission reduction, but there are still the following shortcomings: (1) the evaluation indicator system of carbon emission needs to be further enriched. So the research viewpoints of many scholars above was synthesized, the indicator system studied by them was summarized and generalized, and five specific indicators were finally formed. (2) The methods of evaluation and prediction on carbon emission needs to be further integrated. For example, matter-element model is widely used in ecological environment evaluation, water resources carrying capacity evaluation and land ecological level evaluation, and it has a good evaluation effect^24–26^, but it is rarely used in the fields of carbon emission evaluation. In order to enrich the analysis methods of carbon emission, matter-element model is used to analyze the regional carbon emission combined with gray prediction model.

As a member of the Yangtze River Delta city cluster, Huaibei City is rich in coal resources and has a high level of carbon emission. Carbon emission reduction in the city has attracted wide attention from society and the public. Based on the numerous researches of carbon emission reduction, this study on the carbon emission in Huaibei City is representative and can provide theoretical and policy references for the low-carbon development of other similar cities in China.

Therefore, an indicator system of carbon emission including Gross domestic product (GDP), Energy intensity, Urbanization, Proportion of secondary industry, and Population was used to evaluate and predict the carbon emission of Huaibei city in China based on matter-element model and gray prediction model.

## Materials and methods

### Study area

Huaibei, referred to as “Huai”. Located in northern Anhui Province, between east longitude 116° 23ʹ–117° 02ʹ, north latitude 33° 16ʹ–34° 14ʹ. It is 150 km long from north to south and 50 km wide from east to west, with a total area of 2802 square kilometers. It is located in the hinterland of East China, at the intersection of Jiangsu, Shandong, Henan and Anhui provinces, with Xiao County to the north, Mengcheng to the south, Suzhou to the east, Guoyang and Henan Yongcheng to the west.

As one of the prefecture-level cities in Anhui Province, it has jurisdiction over one county and three districts. By the end of 2022, the total resident population and urbanization rate were 1.974 million and 64.78% respectively, and ranking 13th and 5th in Anhui Province respectively. It is a national coal base and its economy is dominated by coal industry. In 2022, the cumulative value of Huaibei’s regional GDP was 130.28 billion yuan, an increase of 7.98 billion yuan over the same period in 2021, with a real growth of 0.2%. The three industrial structure of Huaibei City is 6.8:43.8:49.4, the pace of urban transformation has become more solid.

### Comprehensive assessment of impact factors

#### Construction of indicator system

Based on the selection of research indicators by relevant scholars and the core research points of this study, the indicator system is constructed based on the level of economic and social development (GDP, urbanization rate), population size (population), technical factor (energy intensity) and industrial structure (the proportion of secondary industry). The specific is shown in Table 1.

**Table 1:** Carbon emission development evaluation indicator system of Huaibei.

The target layer	Indicator layer	Symbol	Trend
Evaluation of carbon emission development in Huaibei City	GDP (one hundred million yuan)	C1	+
	Energy intensity (ton standard coal/ten thousand yuan)	C2	+
	Urbanization (%)	C3	+
	Proportion of secondary industry (%)	C4	+
	Population (ten thousand)	C5	−

The indicator tends to be “+”, indicating that the larger the indicator, the more conducive to development; The indicator tends to be “−”, indicating that the larger the indicator, the more inhibited the development.

#### Entropy weight method

The entropy weight method is objective, and its calculation steps are given as follows^27–29^.

First, different trend indicators are standardized in order to facilitate the comparison (shown in Eqs. (1), (2)).

1 $$  {\text{Positive indicator}}:\;y_{{ij}}  = \frac{{{\text{[x}}_{{{\text{ij}}}}  - {\text{min}}\left( {{\text{x}}_{{\text{j}}} } \right){\text{]}}}}{{{\text{[max}}\left( {{\text{x}}_{{\text{j}}} } \right) - {\text{min}}\left( {{\text{x}}_{{\text{j}}} } \right)}},  $$

2 $$  {\text{Negative indicator}}:\;y_{{ij}}  = \frac{{{\text{[max}}\left( {{\text{x}}_{{\text{j}}} } \right) - {\text{x}}_{{{\text{ij}}}} {\text{]}}}}{{{\text{[max}}\left( {{\text{x}}_{{\text{j}}} } \right) - {\text{min}}\left( {{\text{x}}_{{\text{j}}} } \right)}}. $$

Then, the information entropy and the weight of each indicator were obtained by Eqs. (3)–(5).

3 $$  e_{j}  =  - \frac{1}{{\ln m}}\sum\limits_{{i = 1}}^{m} {f_{{ij}} \ln f_{{ij}} } ,  $$

4 $$  f_{{ij}}  = \frac{{y_{{ij}}  + 1}}{{\sum\nolimits_{{i = 1}}^{m} {\left( {y_{{ij}}  + 1} \right)} }}, $$

5 $$ \omega _{j}  = \frac{{1 - e_{j} }}{{n - \sum\nolimits_{{j = 1}}^{n} {e_{j} } }}, $$

where, when $f_{ij} = 0$, let $f_{ij}\,\ln f_{ij} = 0$, and $0 \le W_{j} \le 1$, $\sum\nolimits_{j = 1}^{n} {W_{j}} = 1$.

#### Analytic hierarchy process method

The analytic hierarchy process is a subjective weight method, and its calculation steps are given as follows^30^.

First, the judgment matrix $A$ is constructed according to the relative importance of each indicator (Eq. 6).

6 $$  A = \left( {a_{{ij}} } \right)_{{n \times n}}  = \left( {\begin{array}{*{20}c}    {a_{{11}} } &  \cdots  & {a_{{1n}} }  \\     \vdots  &  \ddots  &  \vdots   \\    {a_{{n1}} } &  \cdots  & {a_{{nn}} }  \\   \end{array} } \right). $$

Second, the weights of each indicator are obtained according to Eq. (7), and the weights are standardized by Eq. (8).

7 $$ W^{\prime}_{\left( {{\text{1}} \times {\text{n}}} \right)} = \left( {\begin{array}{*{20}c}    {\omega _{{{{1^{\prime}}}}} }  \\    {\omega _{{{{2^{\prime}}}}} }  \\     \vdots   \\    {\omega _{{n - 1^{\prime}}} }  \\    {\omega _{{n^{\prime}}} }  \\   \end{array} } \right) = \left( {\begin{array}{*{20}c}    {\sqrt[n]{{\mathop \Pi \nolimits_{{j = 1}}^{n} a_{{1j}} }}}  \\    {\sqrt[n]{{\mathop \Pi \limits_{{j = 1}}^{n} a_{{2j}} }}}  \\     \vdots   \\    {\sqrt[n]{{\mathop \Pi \nolimits_{{j = 1}}^{n} a_{{n - 1,j}} }}}  \\    {\sqrt[n]{{\mathop \Pi \nolimits_{{j = 1}}^{n} a_{{nj}} }}}  \\   \end{array} } \right), $$

8 $$  W = \left( {\begin{array}{*{20}c}    {\frac{{\omega _{{{{1^{\prime}}}}} }}{{{{W^{\prime}}}}}}  \\    {\frac{{\omega _{{{{2^{\prime}}}}} }}{{{{W^{\prime}}}}}}  \\     \vdots   \\    {\frac{{\omega _{{{\text{n}} - {{1^{\prime}}}}} }}{{{{W^{\prime}}}}}}  \\    {\frac{{\omega _{{{{n^{\prime}}}}} }}{{{{W^{\prime}}}}}}  \\   \end{array} } \right) = \left( {\begin{array}{*{20}c}    {\omega _{{\text{1}}} }  \\    {\omega _{{\text{2}}} }  \\     \vdots   \\    {\omega _{{{\text{n}} - {\text{1}}}} }  \\    {\omega _{{\text{n}}} }  \\   \end{array} } \right).  $$

Finally, the consistency test is performed according to Eqs. (9) and (10) and Table 2.

9 $$ C.I. = \frac{{\lambda_ {\text{max}} - {1}}}{{{\text{n}} - {1}}}, $$

10 $$ C.R. = \frac{C.I.}{{R.I.}}. $$

**Table 2:** Consistency test standard.

n	1	2	3	4	5	6	7	8	9	10	11
R.I	0	0	0.58	0.90	1.12	1.24	1.32	1.41	1.45	1.49	1.51

If *C.R.* < 0.1, the judgment matrix is considered to pass the consistency test.

#### Game theory weighting method

Game theory can play an important role in weighting calculations, and more accurate combined weights can be obtained by assigning a reasonable proportion to subjective and objective weights^31^. That is, the objective weight determined by the entropy weight method $W_{1}$ as a party of the game, the subjective weight determined by the analytic hierarchy process method $W_{2}$ as the other side of the game, then the combination weight of the two sides of the game to reach the equilibrium state is the optimal combination weight. The specific steps are given as follows.

First, the combination weight $W^{\prime}$ is obtained by Eq. (11).

11 $$ W^{\prime} = \alpha W_{1} + \beta W_{2} , $$

where, $\alpha$ and $\beta$ represent the weight distribution coefficients of $W_{1}$ and $W_{2}$ respectively. $W_{1}$ denote the indicator weight obtained by analytic hierarchy process method and $W_{2}$ denote the indicator weight obtained by entropy weight method.

Second, the optimal weight coefficient is calculated according to the normal equation (Eq. 12).

12 $$ \left( {\begin{array}{*{20}c} {\left( {W_{1} ,W_{1} } \right)} & {\left( {W_{1} ,W_{2} } \right)} \\ {\left( {W_{2} ,W_{1} } \right)} & {\left( {W_{2} ,W_{2} } \right)} \\ \end{array} } \right)\left( {\begin{array}{*{20}c} \alpha \\ \beta \\ \end{array} } \right) = \left( {\begin{array}{*{20}c} {\left( {W_{1} ,W_{1} } \right)} \\ {\left( {W_{2} ,W_{2} } \right)} \\ \end{array} } \right). $$

Finally, the comprehensive weight is obtained by Eq. (13).

13 $$ W = \alpha_{1} W_{1} + \alpha_{2} W_{2} , $$

where $$\alpha_{1} = \frac{\alpha }{\alpha + \beta },\;\alpha_{2} = \frac{\beta }{\alpha + \beta }.$$

### Matter-element model

Matter-element model is an effective method to study matter-element and its variation rules and solve incompatible problems. It is often used in the comprehensive evaluation research of a certain system^32,33^. The specific steps are given as follows.

First, the matter-element system of carbon emission development should be constructed by Eq. (14).

14 $$ R = \left| {\begin{array}{*{20}c} {\text{Carbon }} & {GDP} & {v_1} \\ {{\text{emission}}} & {\text{Energy intensity}} & {v_2} \\ {\text{ development}} & {{\text{Urbanization}}} & {v_3} \\ {{\text{in}}} & {\text{Proportion of secondary industry}} & {v_4} \\ {{\text{Huaibei}}} & {{\text{Population}}} & {v_5} \\ \end{array} } \right|. $$

Second, the classical domain $R\sigma {j} = (N_{\sigma j},W_{i},V_\sigma )$ and nodal domain $Rp = (N_{p},W_{n},V_{p})$ are determined by Eqs. (15) and (16) respectively.

15 $$ R_{\sigma j} = (N_{\sigma j},W_{i},V_{\sigma} ) = \left| {\begin{array}{*{20}c} {N_{\sigma j}} & {w_{1}} & {(a_{\sigma j1},b_{\sigma j1})} \\ {} & {w_2} & {(a_{\sigma j2},b_{\sigma j2})} \\ {} & {...} & {...} \\ {} & {w_n} & {(a_{\sigma jn},b_{\sigma jn})} \\ \end{array} } \right|, $$

where $R_{\sigma j}$ is the matter element of the classical domain, $N_{\sigma j}$ is the $j$ evaluation level, $W_i$ is the $i$ evaluation indicator, and $(a_{\sigma ji},b_{\sigma ji})$ is the magnitude range of the corresponding evaluation level $j$.

16 $$ Rp = (N_p,W_n,V_p) = \left| {\begin{array}{*{20}c} {N_p} & {w_1} & {(a_{p1},b_{p1})} \\ {} & {w_2} & {(a_{p2},b_{p2})} \\ {} & {...} & {...} \\ {} & {w_n} & {(a_{pn},b_{pn})} \\ \end{array} } \right|, $$

where $R_p$ is the matter element of the node domain, $N_p$ is the evaluation level, and $(a_{pn},b_{pn})$ is the value range of $W_n$ corresponding to the matter element of the node domain.

Third, the evaluation indicator correlation degree function and correlation degree are determined. The correlation degree function $K(x)$ of the carbon emission development evaluation indicator and the real function distance can be obtained by Eqs. (17) and (18).

17 $$ K(x) = \left\{ \begin{gathered} \frac{ - \rho (X,X_o)}{{\left| {X_o} \right|}}(X \in X_o) \hfill \\ \frac{\rho (X,X_o)}{{\rho (X,X_p) - \rho (X,X_o)}}(X \notin X_o) \hfill \\ \end{gathered} \right., $$

18 $$ \left\{ \begin{gathered} \rho (X,X_o) = = \left| {X - \frac{1}{2}(a_o + b_o)} \right| - \frac{1}{2}(b_o - a_o) \hfill \\ \rho (X,X_p) = = \left| {X - \frac{1}{2}(a_p + b_p)} \right| - \frac{1}{2}(b_p - a_p) \hfill \\ \end{gathered} \right.. $$

Finally, the comprehensive correlation degree and evaluation grade can be obtained by Eq. (19).

19 $$ K_j(N_x) = \sum\limits_{i = 1}^{N} {{W_i}{K_j}(xi)} , $$

where ${K_j}(x_i)$ is the rating level and $W_i$ is the weight of the $i$ indicator. The maximization principle $K_j = \max {K_j}(N_x)$ is adopted to determine the evaluation object’s belonging grade, which means that the evaluated object $N_x$ belongs to the evaluation grade $j$. Further, level I, II, III and IV correspond to safe, relatively safe, less secure and insecure respectively.

### Gray prediction method

The gray prediction method is a method to predict the gray system^34^. And the prediction accuracy is higher under the premise of passing the residual test^35^.

First, the adjacent value is obtained according to Eqs. (20) and (21).

20 $$ z^{\left( 1 \right)} \left( k \right) = \alpha x^{\left( 1 \right)} \left( k \right) + \left( {1 - \alpha } \right)x^{\left( 1 \right)} \left( {k - 1} \right),k = 1,2, \ldots ,n, $$

21 $$ x^{\left( r \right)} \left( k \right) = \sum\limits_{i = 1}^{k} {x^{{\left( {r - 1} \right)}} \left( i \right)} ,k = 1,2, \ldots ,n,r \ge 1, $$

where $\alpha = 0.5$, which is called equal weight neighborhood generating the number.

Second, the gray differential equation model and data matrix are constructed in Eqs. (22) and (23).

22 $$ d\left( k \right) + az^{\left( 1 \right)} \left( k \right) = b, $$

23 $$ U = \left( {\begin{array}{*{20}c} a \\ b \\ \end{array} } \right),\;B = \left( {\begin{array}{*{20}c} { - z^{\left( 1 \right)} \left( 2 \right)} & 1 \\ { - z^{\left( 1 \right)} \left( 3 \right)} & 1 \\ \vdots & \vdots \\ { - z^{\left( 1 \right)} \left( n \right)} & 1 \\ \end{array} } \right),\;Y = \left( {\begin{array}{*{20}c} {x^{\left( 0 \right)} \left( 2 \right)} \\ {x^{\left( 0 \right)} \left( 3 \right)} \\ \vdots \\ {x^{\left( 0 \right)} \left( n \right)} \\ \end{array} } \right), $$

where $d\left( k \right)$ is the gray derivative, $a$ is the development coefficient, $z^{\left( 1 \right)} \left( k \right)$ is the whitening background value, and $b$ is the gray action. Besides, $Y = BU$ and $U = \left( {B^{T} B} \right)^{ - 1} B^{T} Y$.

Third, the corresponding function of the time series is got, and the sequence of predicted numbers of the accumulated generation is obtained by IAGO (shown in Eqs. (24), (25)).

24 $$ x^{\left( 1 \right)} \left( k \right) = \left( {x^{\left( 0 \right)} \left( 1 \right) - \frac{b}{a}} \right)e^{{ - a\left( {k - 1} \right)}} + \frac{b}{a},k = 1,2, \ldots ,n, $$

25 $$ x^{\left( 0 \right)} \left( k \right) = x^{\left( 1 \right)} \left( k \right) - x^{\left( 1 \right)} \left( {k - 1} \right),k = 1,2, \ldots ,n. $$

Finally, the residual test is performed on predicted and actual values by Eq. (26).

26 $$ \varepsilon \left( k \right) = \frac{{x^{\left( 0 \right)} \left( k \right) - \widehat{x}^{\left( 0 \right)} \left( k \right)}}{{x^{\left( 0 \right)} \left( k \right)}},k = 1,2, \ldots ,n, $$

where, when $\left| {\varepsilon \left( k \right)} \right| < 0.2$, the prediction is considered scientific.

## Results

### Indicator weighting calculation

Based on the principle of game theory, the objective weight determined by entropy weight method and the subjective weight determined by analytic hierarchy process method are taken as the two sides of the game. Then the weight that finally reaches the equilibrium state is the optimal combination weight. Furthermore, the optimal combination weight ranking is the indicator importance ranking.

#### Entropy weight analysis results

Combined with Eqs. (1)–(5), the entropy values and weights of each indicator can be obtained (Table 3).

**Table 3:** Weight of each indicator under entropy weight method.

	C1	C2	C3	C4	C5
E_j_	0.8705	0.8531	0.8757	0.7783	0.8830
W_j_	0.1751	0.1987	0.1682	0.2998	0.1583

In Table 3, it can be seen that the importance order of the influence degree of each indicator on carbon emission obtained by entropy weight method is as follows: C4 (the proportion of secondary industry), C2 (energy intensity), C1 (GDP), C3 (urbanization), C5 (population).

#### Analytic hierarchy process analysis results

Combined with Eqs. (6)–(8), the weights of each indicator can be obtained, and the consistency test is passed (Tables 4, 5).

**Table 4:** Weight of each indicator under AHP method.

Indicator	C1	C2	C3	C4	C5
Weight	0.1027	0.3486	0.1014	0.3861	0.0612

**Table 5:** Consistency test result.

$\lambda_{\max }$	5.0727
$C.I.$	0.01817
$R.I.$	1.12
$C.R.$	0.01622

In Table 4, it can be seen that the importance order of the influence degree of each indicator on carbon emission obtained by analytic hierarchy process is as follows: C4 (the proportion of secondary industry), C2 (energy intensity), C1 (GDP), C3 (urbanization), C5 (population).

In Table 5, *C.R.* = 0.01622 < 1 indicates that the above results have passed the consistency test and can be used as the basis for subsequent research.

#### Game theory weighting analysis results

Combined with Eqs. (11)–(13) in order to reduce the error, the comprehensive weights of each indicators can be obtained (Table 6).

**Table 6:** Weight of each indicator under the weighting method of game theory.

Indicator	C1	C2	C3	C4	C5
Weight	0.1565	0.2372	0.1510	0.3220	0.1333

In Table 6, it is obtained by the weighting method of game theory that $\alpha_{1} = 0.2573,\alpha_{2} = 0.7427$. Furthermore, the ranking of indicators importance is as follows: C4 (the proportion of secondary industry), C2 (energy intensity), C1 (GDP), C3 (urbanization), C5 (population).

### Evaluation analysis of impact factors

**Figure 1:**
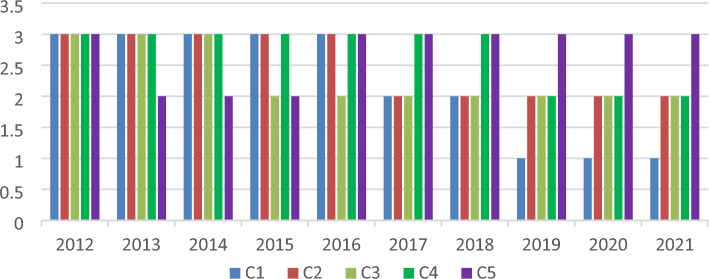
Changes of safety level of each indicator in Huaibei from 2012 to 2021.

Based on the perceptive of Eqs. (14)–(16), the classical domain range of the matter-element evaluation model is constructed (Table 7). Then, combined with Eqs. (17) and (18), the correlation degree of each indicator from 2012 to 2021 are got (Table 8). The corresponding carbon emission safety level changes of each indicator are obtained (Fig. 1). Furthermore, the comprehensive correlation degree of each year are got by Eq. (19) (Table 9).

**Table 7:** Classical domain of matter-element model evaluation indicator.

Indicator	I (safe)	II (relatively safe)	III (less secure)	IV (insecure)
C1	1000–1200	800–1000	600–800	0–600
C2	0–2	2–4	4–6	6–8
C3	70–100	60–70	40–60	0–40
C4	0–30	30–50	50–70	70–100
C5	210–215	215–217	217–219	219–224

The criteria for dividing the classical domain interval are determined according to the minimum and maximum values of the average of the annual historical information of each assessment indicator. I(safe), II(relatively safe), III(less secure), IV(insecure) respectively represents the carbon emission safety status of the indicator. Among them, with the change of levels I to II to III to IV, the promoting effect of the evaluated indicators on carbon emission gradually increases.

**Table 8:** Correlation degree of indicators in Huaibei from 2012 to 2021.

	I	II	III	IV
2012				
C1	− 0.0513	− 0.1025	0.1025**	− 0.5000
C2	− 0.1548	− 0.3097	0.3097**	− 0.5000
C3	− 0.1795	− 0.5000	0.1400**	− 0.1400
C4	− 0.0969	− 0.1938	0.1938**	− 0.5000
C5	− 0.2500	− 0.5000	0.5000**	− 0.5000
2013				
C1	− 0.2453	− 0.5000	0.4815**	− 0.4815
C2	− 0.2461	− 0.5000	0.4845**	− 0.4845
C3	− 0.1154	− 0.5000	0.0750**	− 0.0750
C4	− 0.0689	− 0.1378	0.1378**	− 0.5000
C5	–	0.0000**	0.0000	0.0000
2014				
C1	− 0.1721	− 0.5000	0.2625**	− 0.2625
C2	− 0.2383	− 0.5000	0.4554**	− 0.4554
C3	− 0.0192	− 0.5000	0.0100**	− 0.0100
C4	− 0.0851	− 0.1702	0.1702**	− 0.5000
C5	–	0.0000**	0.0000	0.0000
2015				
C1	− 0.1418	− 0.5000	0.1980**	− 0.1980
C2	− 0.2157	− 0.5000	0.3795**	− 0.3795
C3	− 0.0800	0.0800**	− 0.5000	− 0.0370
C4	− 0.2347	− 0.4694	0.4694**	− 0.5000
C5	− 0.5000	0.5000**	− 0.5000	− 0.2500
2016				
C1	0.0050	− 0.5000	0.0050**	− 0.0050
C2	0.0913	− 0.5000	0.1117**	− 0.1117
C3	0.2100	0.2100**	− 0.5000	− 0.0868
C4	0.1940	− 0.5000	0.3169**	− 0.3169
C5	0.0000	–	0.0000**	0.0000
2017				
C1	− 0.5000	0.3550**	− 0.3550	− 0.1775
C2	− 0.1436	0.1436**	− 0.5000	− 0.1116
C3	− 0.3600	0.3600**	− 0.5000	− 0.1324
C4	− 0.2356	− 1.1783	0.4456**	− 0.4456
C5	0.0000	− 1.0000	0.0000**	0.0000
2018				
C1	− 0.5000	0.0740**	− 0.0740	− 0.0370
C2	− 0.3384	0.3384**	− 0.5000	− 0.2018
C3	− 0.5000	0.4900**	− 0.4900	− 0.1633
C4	− 0.1624	− 1.0962	0.2406**	− 0.2406
C5	− 0.2500	− 1.0046	0.5000**	− 0.5000
2019				
C1	0.3895**	− 0.5000	− 0.2189	− 0.1402
C2	− 0.5000	0.4480**	− 0.4480	− 0.2240
C3	− 0.5000	0.4100**	− 0.4100	− 0.1367
C4	− 0.3634	0.3634**	− 0.5000	− 0.2105
C5	0.0000	0.0000	0.0000**	–
2020				
C1	0.3449**	− 0.3822	− 0.1935	− 0.4414
C2	− 0.1381	0.5933**	− 0.6486	− 0.5021
C3	− 0.0239	0.0700**	− 0.5783	− 0.0628
C4	− 0.0658	0.3372**	− 0.2583	− 0.5546
C5	–	–	0.0833**	–
2021				
C1	0.2696**	− 0.4484	− 0.2377	− 0.2680
C2	− 0.1984	0.3640**	− 0.5006	− 0.3741
C3	− 0.0938	0.2278**	− 0.1929	− 0.0475
C4	− 0.1265	0.3069**	− 0.4002	− 0.4800
C5	–	–	0.1389**	–

Marked with “**” indicates that the indicator is at this level, and marked with “*” indicates that the indicator is transformed to this level.

**Table 9:** Comprehensive correlation degree summary of Huaibei from 2012 to 2021.

Year	I	II	III	IV	Level
K_j_ (2012)	− 0.1421	− 0.3073	0.2485**	− 0.4412	III
K_j_ (2013)	− 0.1279	− 0.3028	0.2273**	− 0.3318	III
K_j_ (2014)	− 0.1029	− 0.3121	0.1831**	− 0.2782	III
K_j_ (2015)	− 0.2391	− 0.2111	0.0679**	− 0.3034	III
K_j_ (2016)	0.1087	− 0.2914	0.0322**	− 0.1280	III
K_j_ (2017)	− 0.2396	− 0.3786	− 0.1097*	− 0.2019	Transformation to level III
K_j_ (2018)	− 0.3251	− 0.3449	− 0.0267*	− 0.2339	Transformation to level III
K_j_ (2019)	− 0.2163	0.1728**	− 0.3347	− 0.1500	II
K_j_ (2020)	− 0.1133	− 0.3665	0.2043	− 0.3808	III
K_j_ (2021)	− 0.1398	− 0.3240	0.2970	− 0.3373	III

Marked with “**” indicates that the indicator is at this level, and marked with “*” indicates that the indicator is transformed to this level.

Based on the changes in the carbon emission safety level of each indicator in Table 8 and Fig. 1, the following conclusions can be drawn:

1. The carbon emission safety level of energy intensity, secondary industry, GDP, and urbanization have been improved to varying degrees, and the safety level of GDP has the largest improvement. The carbon emission safety level of the population fluctuates but remains at level III (less secure).
2. The level of carbon emission safety level of the five indicators is not synchronized. Among them, the carbon emission safety level of the population improved in 2013 at the earliest and then decreased in 2016, the secondary industry safety level increased in 2019, the GDP safety level increased in 2017 and 2019, the energy intensity safety level increased in 2017, and the urbanization safety level increased in 2015.

According to the summary of comprehensive correlation degree and carbon emission safety level of Huaibei from 2012 to 2021 in Table 9, it can be seen that the change of its safety level can be divided into four stages:

1. From 2012 to 2016: correlation degree value $k_{{j\left( {w2016} \right)}} < k_{{j\left( {w2015} \right)}} < k_{{j\left( {w2014} \right)}} k_{{j\left( {w2013} \right)}} < k_{{j\left( {w2012} \right)}}$, indicating that the carbon emission safety level of Huaibei decreased gradually from 2012 to 2016.
2. From 2017 to 2018: correlation degree value $k_{{j\left( {w2018} \right)}} < k_{{j\left( {w2017} \right)}} < 0$, indicating that the carbon emission safety level of Huaibei in 2018 was lower than that in 2017, and all of them were converted to level III (less secure).
3. In 2019: the maximum value of 0.1728 in 2019 belongs to level II (relatively safe).
4. From 2020 to 2021: correlation degree value $k_{{j\left( {w2021} \right)}} > k_{{j\left( {w2020} \right)}}$, indicating that the carbon emission safety level in 2020 and 2021 has decreased compared with that in 2019, but it is still in the process of slow improvement.

### Prediction analysis of impact factors

Combined with the gray prediction model algorithm and residual test (Eqs. (20)–(26)), it is proven to be scientific and can be used for subsequent data prediction. Then the correlation degree of five indicators (Table 10) and comprehensive correlation degree in the carbon emission safety level evaluation system of Huaibei in 2022–2030 (Table 11) are obtained respectively.

**Table 10:** Correlation degree prediction of indicators in Huaibei from 2022 to 2030.

	I	II	III	IV
C1				
2022	0.0113**	− 0.0782	− 0.0309	− 0.0237
2023	0.0204**	− 0.0782	− 0.0422	− 0.0343
2024	0.0301**	− 0.0782	− 0.0496	− 0.0419
2025	0.0406**	− 0.0782	− 0.0548	− 0.0476
2026	0.0519**	− 0.0782	− 0.0586	− 0.0521
2027	0.0642**	− 0.0782	− 0.0616	− 0.0556
2028	0.07732**	− 0.0782	− 0.0639	− 0.0585
2029	0.0650**	− 0.0650	− 0.0532	− 0.0487
2030	0.0497*	− 0.0497	− 0.0406	− 0.0331
C2				
2022	− 0.1186	0.0467**	− 0.0467	− 0.0234
2023	− 0.1186	0.0229**	− 0.0229	− 0.0114
2024	− 0.1186	0.0010**	− 0.0010	− 0.0005
2025	0.0190**	− 0.1187	− 0.0164	− 0.0088
2026	0.0373**	− 0.1187	− 0.0284	− 0.0161
2027	0.0541**	− 0.1187	− 0.0372	− 0.0220
2028	0.0695**	− 0.1187	− 0.0438	− 0.0269
2029	0.0836**	− 0.1187	− 0.0490	− 0.0309
2030	0.096**	− 0.1187	− 0.0532	− 0.0343
C3				
2022	0.0016**	− 0.0755	− 0.0044	− 0.0015
2023	0.0089**	− 0.0755	− 0.0197	− 0.0079
2024	0.0163**	− 0.0755	− 0.0297	− 0.0134
2025	0.0239**	− 0.0755	− 0.0368	− 0.0182
2026	0.0317**	− 0.0755	− 0.0420	− 0.0223
2027	0.0396**	− 0.0755	− 0.0462	− 0.0260
2028	0.0477**	− 0.0755	− 0.0494	− 0.0292
2029	0.0559**	− 0.0755	− 0.0521	− 0.0321
2030	0.0644**	− 0.0755	− 0.0543	− 0.0347
C4				
2022	− 0.1533	0.1533**	− 0.1610	− 0.0785
2023	− 0.1610	0.1312**	− 0.1312	− 0.0656
2024	− 0.1610	0.0959**	− 0.0959	− 0.0479
2025	− 0.1610	0.0625**	− 0.0625	− 0.0313
2026	− 0.1610	0.0311**	− 0.0311	− 0.0156
2027	− 0.1610	0.0015**	− 0.0015	− 0.0008
2028	0.0176**	− 0.1610	− 0.0226	− 0.0122
2029	0.0351**	− 0.1610	− 0.0397	− 0.0226
2030	0.0516**	− 0.1610	− 0.0522	− 0.0312
C5				
2022	− 0.0318	− 0.0430	− 0.0666	0.0347**
2023	− 0.0000	− 0.0477	− 0.0666	0.0479**
2024	0.0000	− 0.0508	− 0.0666	0.0611**
2025	− 0.0375	− 0.0458	− 0.0589	0.0589**
2026	− 0.0290	0.0000	− 0.0456	0.0456**
2027	− 0.0205	− 0.0251	− 0.0322	0.0322**
2028	− 0.0120	− 0.0146	− 0.0188	0.0188**
2029	− 0.0034	− 0.0042	− 0.0054	0.0000**
2030	0.0052	0.0063	0.0081**	0.0000

Note: *P < 0.1, **P < 0.05, ***P < 0.01.

**Table 11:** Comprehensive correlation prediction of Huaibei in 2022–2030.

Year	I	II	III	IV	Level
K_j_ (2022)	− 0.2908	0.0032**	− 0.3097	− 0.0924	Level II
K_j_ (2023)	− 0.2504	− 0.0473*	− 0.2826	− 0.0714	Conversion to level II
K_j_ (2024)	− 0.2332	− 0.1076	− 0.2428	− 0.0427*	Conversion to level IV
K_j_ (2025)	− 0.1150	− 0.2556	− 0.2294	− 0.0470*	Conversion to level IV
K_j_ (2026)	− 0.0691	− 0.2412	− 0.2058	− 0.0605*	Conversion to level IV
K_j_ (2027)	− 0.0237*	− 0.2959	− 0.1786	− 0.0722	Conversion to level I
K_j_ (2028)	0.2001**	− 0.4480	− 0.1986	− 0.1080	Level I
K_j_ (2029)	0.2362**	− 0.4243	− 0.1993	− 0.1344	Level I
K_j_ (2030)	0.2673**	− 0.3985	− 0.1923	− 0.1333	Level I

In Table 10, from 2022 to 2030, GDP (C1) and urbanization (C3) show a stable state of level I (safe), energy intensity (C2), and the proportion of secondary industry (C4) gradually increased from level II (relatively safe) to level I (safe), only the population decreased to level IV (insecure) and then converted to level III (less secure). This indicates that GDP (C1), urbanization (C3), proportion of secondary industry (C4), and energy intensity (C2) all have positive effects on the improvement of carbon emission safety level, and population (C5) has become the main factor restricting the development of carbon emission in Huaibei, which needs to be paid more attention to.

In Table 11, the development trend of carbon emission in Huaibei from 2022 to 2030 can be obtained:

1. In 2022: the maximum value of the comprehensive correlation degree belongs to level II (relatively safe), and there is no change compared with the carbon emission safety level of 2021 studied above.
2. In 2023: the maximum value of the comprehensive correlation degree is converted to level II (relatively safe), indicating that the carbon emission safety level gradually declines.
3. From 2024 to 2026: the comprehensive correlation degree value $k_{{j\left( {w2026} \right)}} < k_{{j\left( {w2025} \right)}} < k_{{j\left( {w2024} \right)}} < 0$, indicating that the carbon emission safety level will be declining during 2024–2026.
4. In 2027: the comprehensive correlation degree is transformed to level I (safe), and the status of carbon emission safety level evaluation system is gradually improved.
5. From 2028 to 2030: $k_{{j\left( {w2028} \right)}} < k_{{j\left( {w2029} \right)}} < k_{{j\left( {w2030} \right)}}$, indicating that the carbon emission development evaluation level from 2028 to 2030 was gradually improved and stable at level I (safe).

## Discussion

The indicators including Gross domestic product (GDP), energy intensity, urbanization, the proportion of secondary industry, and population are taken as analysis variables in this study. Then, the combination weighting method, matter-element model, and gray prediction model are used to evaluate and predict the carbon emission in Huaibei City. Finally, the following views are formulated.

### Population is the key influencing factor of carbon emission, and its performance is obvious

The comprehensive weight of the population indicator is 0.13 by using the combination weighting method. Meanwhile, in the matter-element model and gray prediction model evaluation and prediction respectively, the carbon emission safety level of the population showed changes from less secure (2012) to relatively safe (2013–2015) to less secure (2016–2021) to insecure (2022–2029) to less secure (2030). Although the carbon emission safety level increased in the middle years, it still showed a downward trend and stayed at level III (less secure) at the end, which highlights the restrictive role of it. Besides, some studies have proved that although population aging causes many problems that are not conducive to economic growth, it reduces carbon emission and promotes the development of the low-carbon economy through the economic hindrance effect, technological progress effect, and industrial structure effect^36^. Meanwhile, other scholars have proved that population growth and its impact on carbon emission reduction promote the low-carbon development of China’s economy, so population growth is a factor that must be considered^37,38^.

### Industrial structure is a non-negligible influencing factor of carbon emission

The proportion of secondary industry in the combination weighting method has the largest weight which is 0.32 and it has a significant and constrained impact on carbon emission. For example, in the matter-element model and gray prediction model evaluation and prediction respectively, the carbon emission safety level of the secondary industry tends to be better. Meanwhile, the impact of industrial structure upgrading on carbon emission reduction has been widely confirmed by scholars at home and abroad^39,40^. As indicated above that to achieve carbon emission reduction in the future, the structural upgrading should be focused on, especially to its integration of technological progress.

### Energy intensity is an important indicator of carbon emission reduction and its practical impact is significant

Based on the combination weighting method, the weight of energy intensity is 0.24, which is an important influencing factor. For example, in the matter-element model and gray prediction model evaluation and prediction respectively, the energy intensity carbon emission safety level has been significantly improved. Relevant studies have showed that the impact of energy structure on carbon emission is significantly positive^41^. It indicates that higher energy consumption will inevitably lead to more serious environmental pollution, and is not conducive to the upgrading of industrial structure, which is in line with the expected hypothesis. Therefore, energy intensity is an important factor restricting carbon emission reduction. Furthermore, the decrease in energy intensity is positively correlated with technological progress^42^. So the technologies of new energy should be strengthened in the future.

### The urbanization process is closely related to the upgrading of household consumption structure and its change characteristics are prominent

In the combination weighting method, the weight of urbanization is 0.15, which indicates that this indicator is an indispensable factor affecting carbon emission. For example, in the matter-element model and gray prediction model evaluation and prediction respectively, the carbon emission safety level of urbanization is in a good situation and steadily improving. Some scholars confirmed that the effectiveness of one-way causality between urbanization and carbon emission in the short term through the estimation results of the vector error correction model, reflecting the importance of urbanization^43^. And the impact of urbanization on carbon emission shows an inverted U-shaped character^44^. So the development strategy of low-carbon should be prioritized to the process of new urbanization.

### GDP is not only an important parameter to measure the level of economic development but also an important factor directly affecting carbon emission

Based on the combination weighting method, the weight of GDP is 0.16, which is one of the important constraint factors of carbon emission. For example, in the matter-element model and gray prediction model evaluation and prediction respectively, the carbon emission safety level of GDP has a good trend. Relevant scholars have found that there is a statistically significant correlation between carbon emission and GDP, which the elasticity coefficient is greater than 1^45^, indicating that carbon emission are sensitive to the changes in GDP. Furthermore, it has also been testified that GDP has the greatest impact on carbon emission^46^, which is consistent with the conclusion in this study. Therefore, it is necessary to find a development path of low-carbon economy, which can not only ensure the growth rate can meet the needs of economic development and social harmony, but also ensure the current requirements of carbon emission reduction.

## Conclusion and suggestion

### Grasping the carbon reduction effect of urbanization on the population to consolidate the new model of low-carbon development

In summary, population is the factor with the lowest influence weight in the evaluation of the safety level of carbon emission, and its safety level has not been significantly improved, but has declined. Population effect is closely related to the urbanization process, and the weight of urbanization is higher than that of population, and its safety level is on the rise. Indicators of population and urbanization should be focused on. In addition, with the acceleration of urbanization, the increase of urban population brings pressure to low-carbon development, but the proposal of new urbanization is a new opportunity for carbon emission reduction. Therefore, in order to improve the carbon emission safety level of population and urbanization, the following suggestions are put forward. For example, carbon emission reduction should be the responsibility and obligation of all sectors, and specific targets should be joint implemented by the government, society, industry and people. Furthermore, more technical talents on carbon emission reduction should be gathered, and its diversified and flexible co-governance and sharing platform should be actively built. Then population resources will become human resources and provide intelligent support for regional carbon emission reduction.

### Identifying the key areas of carbon emission reduction to build a low-carbon emission oriented industrial system

In summary, GDP is the factor with the greatest influence weight in the evaluation of the safety level of carbon emission, and its safety level also shows a significant upward trend. In addition, the proportion of the secondary industry ranks third in the evaluation of the safety level of carbon emission, which has a similar upward trend with GDP. The improvement of the carbon emission safety level of the GDP is closely related to the improvement of the carbon emission safety level of the secondary industry. Therefore, in order to consolidate the role of GDP and the proportion of the secondary industry in the process of carbon emission control, the following suggestions are put forward. For example, it should take carbon emission reduction as the starting point, and implement low-carbon mechanisms in key industries under the premise of achieving high-quality economic development. Furthermore, the low-carbon transformation of traditional industries, the continuous promotion of high-tech industries, and the rapid development of modern service industries and low-carbon agriculture should all be given equal attention and a low-carbon emission oriented industrial system can be built. Then the low-carbon emission oriented industrial system should give full play to the advantages of clean and flexible power, optimize the power system, and expand the development and utilization of clean energy.

### Strengthening the technological innovation of carbon emission reduction to achieve the strategic goal of “dual-carbon”

In summary, the weight of energy intensity in the evaluation of the safety level of carbon emission is second only to that of the secondary industry, and its safety level also shows an increasing trend. Its impact on carbon emission control cannot be ignored. Therefore, in order to strengthen the inhibition effect of energy intensity on carbon emission, the following suggestions are put forward. For example, energy intensity is an important factor in the increase of carbon emission intensity, and the inhibition effect of energy intensity on carbon emission mainly lies in the measure of energy structure optimization. So the use of modern information technology and its intelligent terminal on carbon emission, including blockchain, big data and artificial intelligence, should be accelerated. Then the development path of carbon emission reduction can be explored, the trading market of carbon emission reduction can be improved, and the carbon capture, utilization, and storage technology can be actively promoted and applied. Furthermore, based on the scientific and technological innovation of energy, the overall deployment of relevant de-carbonization, zero carbon, and negative emission technologies can be comprehensively strengthened, the research and development demonstrations of carbon emission reduction can be accelerated. Then the utilization of clean coal technology can be strengthened and the depth and breadth of clean energy utilization can be expanded to achieve the strategic goal of “dual-carbon”.

## Data Availability

All data generated or analyzed during this study are included in this article.
